# Alteration in social interaction and tactile discrimination of juvenile autistic‐like rats following tactile stimulation and whisker deprivation

**DOI:** 10.1002/brb3.2993

**Published:** 2023-04-16

**Authors:** Bi Bi Marzieh Ahmadi, Mohammad Reza Afarinesh, Leila Jafaripour, Vahid Sheibani

**Affiliations:** ^1^ Neuroscience Research Center Institute of Neuropharmacology Kerman University of Medical Sciences Kerman Iran; ^2^ Cognitive Neuroscience Research Center Institute of Neuropharmacology Kerman University of Medical Sciences Kerman Iran; ^3^ Department of Anatomy School of Medicine Dezful University of Medical Sciences Dezful Iran

**Keywords:** autism, barrel cortex, tactile stimulation, texture discrimination, three‐chamber social interaction, whisker deprivation

## Abstract

**Introduction:**

Autism spectrum disorder is a developmental disorder that can affect sensory‐motor behaviors in the valproic acid (Val) rodent model of autism. Although whisker deprivation (WD) induces plastic changes in the cortical neurons, tactile stimulation (TS) during the neonatal period may reverse it. Here, we investigate the interaction effects of TS and WD on behavioral and histologic features of barrel cortex neurons in juvenile Val‐treated.

**Methods:**

Control (CTL, CTL–TS, CTL–WD, and CTL–TS–WD groups) and Val‐treated (Val, Val–TS, Val–WD, and Val–TS–WD groups) rats of both sexes were subjected to behavioral tests of social interaction, and novel texture recognition, and Nissl staining. The TS groups were exposed to sensory stimulation for 15 min, three times/day; moreover, all whiskers in the WD groups were trimmed every other day from postnatal days 1 to 21.

**Results:**

Both prenatal valproic acid administration and postnatal WD decreased the rats’ performance percentage of the Val and CTL–WD groups of both sexes compared with the CTL groups in the social interaction and texture discrimination tests. Following TS, the performance of the Val–TS–WD group increased significantly compared to the Val group (*p* < .05), whereas the performance of the CTL–TS–WD group rescued to the CTL group. Nissl staining results also revealed the neuron degeneration percentage in the barrel field area of the Val and CTL–WD groups was increased significantly (*p* < .05) compared with the CTL group. In this regard, TS decreased the neuron degeneration percentage of the Val–TS–WD and the CTL–TS–WD groups, compared with the CTL group, significantly (*p* < .05).

**Conclusion:**

TS in juvenile male and female rats can act as a modulator and compensate for the behavioral and histological consequences of WD and prenatal valproic acid exposure.

## Graphical abstract

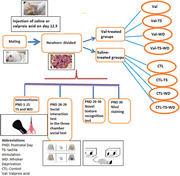

## 1 INTRODUCTION

One of the most important roles in the initial stages of human development is played by somatosensory perception. The sense of touch can be detected by fetuses responding to the touching of the mother's abdomen (Pihko & Lauronen, 2004). It may develop faster than all other sensory systems (Kadić & Predojević, 2012). Newborns immediately use tactile input from the perioral area to stimulate sucking and rooting reflexes necessary for feeding (Kaffman & Meaney, 2007; Muir, 2002). The feedback between a person and his environment that he receives through the sense of touch in infancy and adolescence forms many of his motor skills, social interactions, and communication (Sengupta, 2013). For a considerable period, the sense of touch is the main means of communication between caregivers and infants before learning a language. Ample maternal contact during childhood elicits increased vocalization and smiling in the infant (Stack & Muir, 1992), and reciprocity between infant and mother and the coordination between infant–mother interactions are affected by the frequency of maternal touch (Ferber et al., 2008; Nicholas, 2010).

These reciprocal rhythms of interaction learned in infancy lay the foundation for social communication later in life. Infants also use touch to communicate distress (Moszkowski et al., 2009) and basic needs. Thus, tactile communication serves as a precursor (Nicholas, 2010) to verbal communication, and the patterns formed during this period impact the development of more sophisticated communication skills and a framework for interaction with the family and community. Even in adults, tactile input can enhance the perception of auditory and visual speech (Gick et al., 2008) and is a primary means of nonverbal communication.

Neurological developmental disorders include many childhood conditions in which cognitive, motor, emotional, or social differences are caused by known or probable nervous system functional abnormalities (de Rόiste & Bushnell, 1996; Nithianantharajah & Hannan, 2006). In the early stages of development, contact plays an important role in the areas mentioned above.

Attention deficit hyperactivity disorder (ADHD) has been associated with behavioral activity levels, and somatosensory processing in ADHD has been widely investigated. Tactile and other sensory processing problems are common problems among children with autism spectrum disorder (ASD). Impaired social and communication skills are a core symptom of ADS. Neonatal tactile stimulation (TS) has been found to be an effective treatment for alleviating the emotional and cognitive symptoms of ASD at an early age (Raza et al., 2015; Toda & Fogel, 1993). In ASD, TS is a reliable method of reducing stress and anxiety, cortisol levels (Field et al., 1992), and stereotypic behavior whilst also improving spatial working memory (Escalona et al., 2001; Zhang & Cai, 2008), face‐to‐face interaction, and communication with the caregiver (Ramey & Ramey, 1998). As spine density, dendritic length, and dendritic branching are increased in the amygdala and prefrontal cortex by TS, it has been found that dendritic morphology in these regions is dramatically altered by TS in rats (Raza et al., 2015).

Rodents prenatally exposed to valproic acid show neurodevelopmental delays and neuroanatomical and behavioral alterations similar to those observed in humans with ASD (Raza et al., 2015). Lower body weight and delayed maturation and motor development are reported in valproic acid–treated rats (Itzchak et al., 2011; Schneider & Przewłocki, 2005). These abnormalities can be reversed under the influence of the enriched environment so that body weight gain increases in the enrichment environment (Konkle et al., 2010). An enrichment environment also improves object recognition and stereotyped behaviors in mice (Mesa‐Gresa et al., 2013) such as grooming and rearing. It is assumed that pseudo‐anxious behaviors increase in an animal model of autism (Moy et al., 2008).

In humans, primary somatosensory (SI) areas are stimulated by the actual touch delivered on the skin (Gazzola et al., 2012). In rodents, whiskers are the most important tactile apparatus (Meaney et al., 1988), which is used for experience‐dependent plasticity studies (Shamsizadeh et al., 2007; Sheibani et al., 2010; Sheikhkanloui‐Milan et al., 2010). The array of macro‐whiskers on the snout provides rodents with tactile sensory information relating to the size, shape, and texture of objects in their immediate environment. Rodents can use their whiskers to detect stimuli, distinguish textures, locate objects, and navigate (Meaney et al., 1988). The whiskers are represented in S1 by a set of cellular aggregates in layer 4 of the somatosensory cortex (L4), termed “barrels,” which corresponds one‐to‐one to single whiskers on the snout (Woolsey & Van der Loos, 1970).

Because of the brain's plasticity, enriching experiences should be able to induce considerable change in it. There are indications that motor performance and/or cognition are improved, and normal brain function is radically changed by exposure to environmental stimuli at an early age (Malabou, 2009). Although the positive effect of environmental enrichment has been proved on rats’ behaviors related to tactile discrimination and also on the neural barrel cortex functions in normal rats (Huang et al., 2021) and animal models of autism (Sabzalizadeh et al., 2022), the effects of TS treatment on the tactile and social behavior of prenatal Val‐exposed rats deprived of whiskers are unknown. The present study assessed the effects of TS treatment on social and tactile discrimination behavior and morphology of barrel cortical neurons in the rat model of autism.

## 2 MATERIALS AND METHODS

### 2.1 Animals

*Wistar* rats (200–300 g) that were born and maintained at the animal house of Kerman Neuroscience Research Center were subjected to the present experimental study. All experiments were approved by the Kerman Neuroscience Research Center (ethics approval code: IR.KMU.AH.REC.1400.037) and conducted following the guidelines of the National Institute of Health for the care and maintenance of laboratory animals. The rats were maintained in a controlled environment with 12‐h light/dark cycles and constant temperature (22 ± 2°C) in the animal house with ad libitum access to food and water. Females were housed in groups of two per cage with a single male. The day on which spermatozoa were detected in the vaginal smear was considered gestational day 1 (GD 1). Pregnant rats were exposed to Val (600 mg/kg) or sterile saline (0.9%) solution on 12.5 of embryonic development (Favre et al., 2013; Schneider & Przewłocki, 2005) After birth, the rat pups that were taken from mothers who had received saline on GD 12.5 were divided into control (CTL), TS, whisker deprivation (WD), and TS plus WD (TS–WD) groups. Those whose mothers had received valproic acid on GD 12.5 were divided into valproic acid (Val), valproic acid plus TS (Val–TS), valproic acid plus WD (Val–WD), and valproic acid plus TS and WD (Val–TS–WD) groups. The pups were randomly selected from pregnant mothers (saline‐ or valproic acid–treated) to enter group. To avoid the litter's size effect, a male or female pup was selected from each mother for the behavioral tests and the Nissl staining study. Totally, 112 pups were used in this study. The survival rate of the Val‐exposed pups’ model was 35%, due to spontaneous abortions and severe congenital malformations (Table 1). The rats were subjected to behavioral tests of social interaction and novel texture recognition (NTR) on the 28th–29th and 29th–30th after birthdays, respectively. Moreover, the body weight gain of rats was measured on postnatal days (PNDs) 1 and 30 (Table 2). Tail abnormalities were monitored in the Val‐treated groups.

**TABLE 1:** Grouping of offspring rats whose mothers received saline or valproic acid on the 12.5th day of pregnancy

	After delivery: a pup from each parent is randomly subjected to each group				
Pregnant mothers	*Group*	*Sex*	*Definition*	*Behavioral study (PNDs 28–30)*	*Nissl staining* ^b^ *(PND 30)*
Number of pregnant rats^a^	CTL	Male/Female	The pups had no intervention during the experiment	For saline‐treated *n* = 7/7 For Val‐treated *n* = 7/7 (male/female)	*n* = 3/3 (male/female)
	WD	Male/Female	All whiskers of the rat were trimmed from PNDs 1 to 21	For saline‐treated *n* = 7/7 For Val‐treated *n* = 7/7 (male/female)	*n* = 3/3 (male/female)
	TS	Male/Female	TS was performed from PNDs 1 to 21	For saline‐treated *n* = 7/7 For Val‐treated *n* = 7/7 (male/female)	*n* = 3/3 (male/female)
	TS‐WD	Male/Female	A rat experienced both WD and TS from PND 1 to 21	For saline‐treated *n* = 7/7 For Val‐treated *n* = 7/7 (male/female)	*n* = 3/3 (male/female)

^a^ Ten pregnant females’ mothers received saline and 40 pregnant females’ mothers received valproic acid (Val).

^b^ After behavioral test, the rats were randomly subjected to histological study.

Abbreviations: CTL, control; PND, postnatal day; TS, tactile stimulation; WD, whisker deprivation.

**TABLE 2:** Offspring rats’ body weight at postnatal days (PNDs) 1 and 30

Groups									
		CTL	CTL–WD	CTL–TS	CTL–TS–WD	Val	Val–WD	Val–TS	Val–TS–WD
Body weight at PND 1	Male	6.28 ± 0.28	5.71 ± 0.36	6.57 ± 0.20	5.86 ± 0.26	5.43 ± 0.20	5.43 ± 0.30	6.14 ± 0.26	5.43 ± 0.20
	Female	6.57 ± 0.20	5.71 ± 0.29	5.57 ± 0.30	6.14 ± 0.26	5.43 ± 0.20	5.57 ± 0.37	6.43 ± 0.20	5.57 ± 0.20
Body weight at PND 30	Male	41.7 ± 1.27	35.43 ± 0.84	34.29 ± 1.04	45.71 ± 1.99	38.57 ± 1.74	^c^53.14 ± 2.9	^d^51.57 ± 0.9	^e^59.0 ± 2.51
	Female	44 ± 1.19	^a^35.00 ± 2.3	^b^35.00 ± 1.02	50.43 ± 2.19	38.57 ± 1.21	32.29 ± 2.50	49.14 ± 0.51	38.43 ± 1.74

*Note*: Data are shown as mean ± SEM (*n* = 7). “a” denotes a significant statistical difference between the female–CTL–WD group and the female–CTL group at PND 30. “b” denotes a significant statistical difference between the female–CTL–TS group and the female–CTL group. “c” denotes a significant statistical difference between the male–Val–WD group and the male–Val group at PND 30. “d” denotes a significant statistical difference between the female–Val–TS group and the female–Val group at PND 30. “e” denotes a significant statistical difference between the male–Val–TS–WD group and the male–Val group at PND 30.

Abbreviations: CTL, control; TS, tactile stimulation; WD, whisker deprivation.

### 2.2 Tactile stimulation procedure

The offspring rats were housed together with their mothers until PND 21. The intervention of TS was performed in another test room from head to tail with a soft brush in 15‐min periods at 09:00, 14:00, and 17:00 every day, and the pups were then returned to their mother's cage after every brushing. Brushing was performed from the first day of birth to PND 21 (Raza et al., 2015).

### 2.3 Sensory deprivation procedure

For sensory deprivation (WD) induction, all whiskers (macro vibrissa) were carefully and thoroughly cut from the first day of birth to PND 21 with small sharp scissors every other day. For the groups that received both interventions together, the whiskers were cut first, and then TS was done according to the protocol (Lee et al., 2009; Sheibani et al., 2010).

### 2.4 Three‐chamber social test

The social interaction test was used to determine the social interactions in rats. The maze consists of three chambers (51 × 51 × 20 cm) separated by two walls. First, in the habituation phase, the rat was placed in the empty maze for 10 min. On the second day (phase 1), during the sociability phase, an unfamiliar rat as the “stranger rat” with the same weight, age, and sex was placed inside a wire cage in one of the side chambers (Stranger 1 chamber). An empty cage was placed on the opposite side of the maze (object chamber). The wall between the mazes was then removed, and the test subject was left to examine the three parts of the maze freely for 10 min. After a 5‐min novelty phase, another unfamiliar rat (Stranger 2) was placed in the object chamber under the wire cage, and the subject rat was allowed to explore Strangers 1 and 2 for 10 min (phase 2). Social interaction between animals was recorded by a video camera built into the top of the maze. The social interaction of the subject was defined as the time the subject reached a distance of 2 cm from the wire cages in the side chambers. Social parameters were analyzed by a blind researcher. At the end of each session, the floor and walls of the maze were cleaned with 2% alcohol. The preference index for Stranger 1 as the sociability index was calculated by the formula: exploration time of Stranger 1/(exploration time of Stranger 1 + exploration time of object chamber) × 100.

The preference index for Stranger 2 as the novel social index was calculated by the following formula: exploration time of Stranger 2/(exploration time of Stranger 1 + exploration time of Stranger 2) × 100 (Rad et al., 2022; Rajizadeh et al., 2021).

### 2.5 Novel texture recognition (NTR)

A wooden maze with dimensions of 40 × 50 × 40 cm was used in this experiment. The objects used were cylindrical iron plates with dimensions of 30 × 6 cm (height = 30 and diameter = 6 cm), covered with sandpaper (P220 and P180) with an average particle size of 68 and 82 μm. The objects were placed at equal distances from each other inside the maze. This test was performed on 2 consecutive days at a specified time. One hour before the test, the subject rats were kept in a separate cage. On the first day of the experiment, which was the habituation phase, each rat was allowed to explore the maze for 10 min. In the familiar phrase (phase 1), the subject explored two similar textures (P220) for 10 min. The subject was returned to its cage for 5 min to rest, and then one of the textures was randomly removed and replaced with a new texture (P180). Then, the subject was returned to the maze to explore the textures for 5 min during the test phase (phase 2). A video camera and data recorded all events were analyzed by a blind researcher. Preference index as a criterion of preference for finding novel objects in the NTR test used by the formula:

Exploration time for new texture/(exploration time for the new texture + exploration time for old texture) × 100 (Afarinesh et al., 2020; Sabzalizadeh et al., 2021).

In order to investigate repetitive and anxiety‐like behaviors, the rearing and grooming of rats were evaluated during phases 1 and 2 in both the three‐chamber social interaction and the texture tests.

### 2.6 Tissue preparation and Nissl staining

For studying histological features of the barrel cortex, three pups from each group were randomly selected and were deeply anesthetized with urethane (1.2 g/kg) on PND 30 (Table. 1). The cortex of the right hemisphere was removed and fixed in 10% formalin. Tissue processing was performed with increasing degrees of alcohol for dehydration, and clarification was performed with xylene. Then, the sample was embedded in paraffin for cutting. Cutting started from 2.9 mm after bregma and continued for 1 mm after that. Paraffin block sections with a thickness of 6–7 μm were cut using a microtome. Tissue sections were deparaffinized with xylene, rinsed once with alcohol, stained with cresyl violet solution for 3 min, 100% dehydrated in alcohol, processed in xylene, and so mounted mistreatment entellan. Normal and degenerated nerve cells were counted in six visual fields at ×100 and ×400 magnifications. Then, the percentage of degenerated neurons in every group was calculated (Bigdeli et al., 2019; Pourmobini et al., 2022) as follows:

(The number of degenerated neurons in each group/total neurons) × 100.

### 2.7 Statistical analysis

The data collected from the three‐chamber social and novel texture discrimination tests and histological tests were analyzed using a four‐way ANOVA. If a significant main effect was found by the four‐way ANOVA or the interaction of sex × pre‐treatment × TS × WD, then the main statistical effects of significant variables were interpreted by performing a one‐way ANOVA followed by the Bonferroni post hoc test to find statistical differences. However, pretreatment was defined as the injection of a single dose of valproic acid or saline during the prenatal period. If a significant main effect was found by the four‐way ANOVA or the interaction of sex × pretreatment × TS × WD, then the main statistical effects of significant variables were interpreted by performing a one‐way ANOVA followed by the Bonferroni post hoc test to find statistical differences. The body weight gain data were analyzed using a four‐way repeated measurement ANOVA. Data are shown as mean ± SEM, and *p* < .05 was set as the significance criterion.

## 3 RESULTS

### 3.1 Changes in body weight gain

Repeated measurement of ANOVA (time × pretreatment × sex × TS × WD) of the body weight gain of offspring's rats showed a significant main effect for time [*F*(1,96) = 6.84, *p* = .0001], time × pretreatment [*F*(1, 96) = 35.53, *p* = .0001], time × sex [*F*(1, 96) = 24.97, *p* = .0001], time × TS [*F*(1, 96) = 36.98, *p* = .0001], and time × WD [*F*(1, 96) = 8.94, *p* = .004]. The interaction of time × pretreatment × sex × TS × WD was not significant. More analysis showed that sensory deprivation reduced the percentage of body weight gain (3.35% for males and 5.07% for females) in the CTL–WD groups compared with the CTL groups, which was significant only in females (*p* = .009). Male rats of the Val–WD group also revealed an increase (7.29%) in their body weight compared with the Val group (*p* = .0001) (Table 2).

Exposure to TS decreased the body weight gain by 4.89% in the female rats of the CTL–TS group compared with the female‐CTL group (*p* = .009), whereas it was not significant in males (3.57%). Female rats belonging to the Val–TS group also revealed a significant increase in their body weight gains compared with the Val group (6.14%, *p* = .002), whereas it was not significant in male rats (5.79%).

In both sexes, co‐exposure to TS and WD had no significant statistical changes in the body weight gain of the CTL–TS–WD group (1.79% for males and 2.57% for females) compared with the CTL groups. The body weight gain of the male‐Val–TS–WD group showed a significant increase (*p* = .0001) compared with the Val group (10.21%), but it was not significant in the female rats (Table 2).

### 3.2 Three‐chamber social test

#### 3.2.1 Preference index to Stranger 1 (PIST1)

**FIGURE 1:**
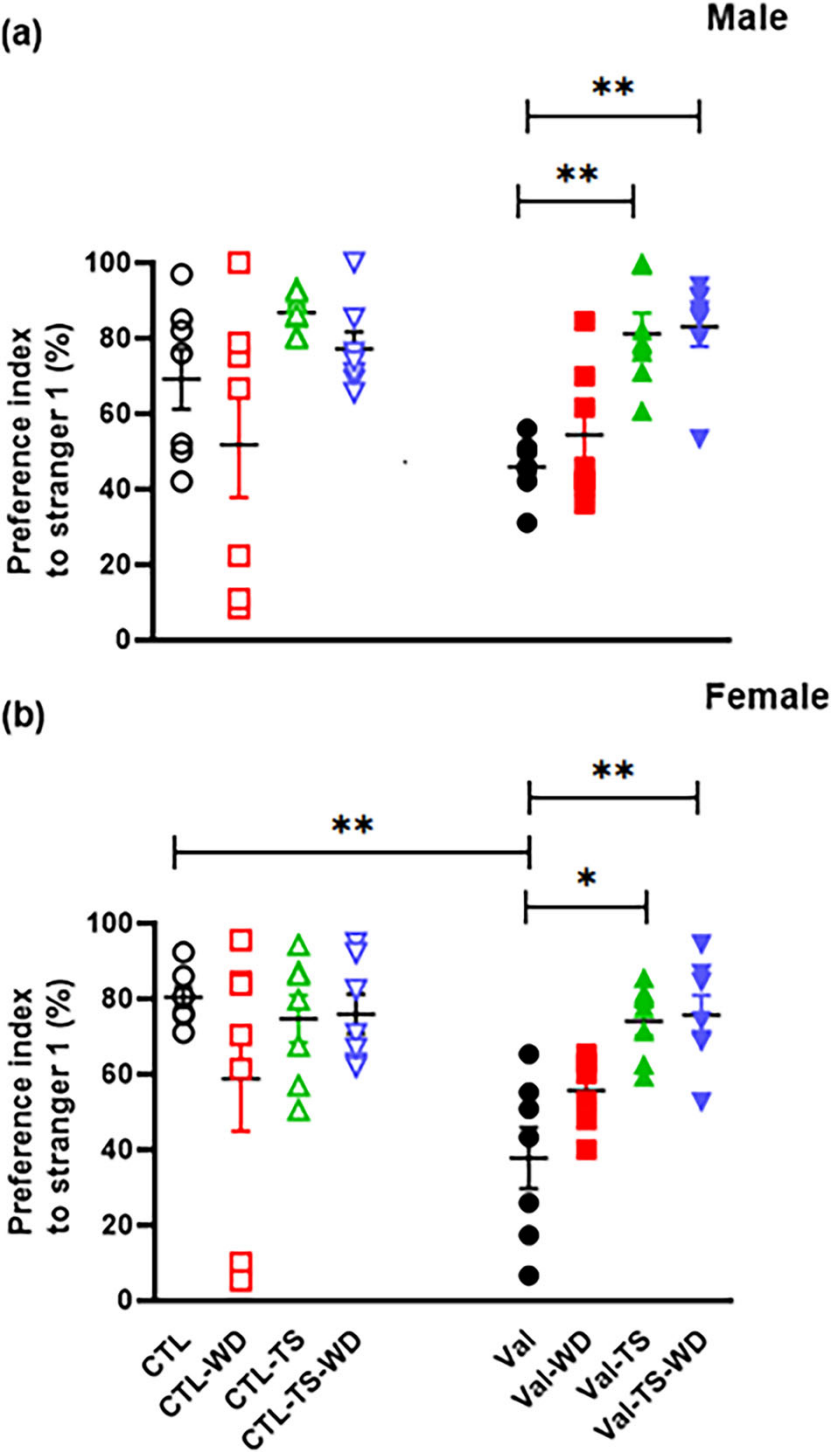
Sociality phase of three‐chamber social test: The graphs show the percentage of the preference index to Stranger 1 (PIST1) to the object chamber in both male (a) and female (b). The rats were prenatally treated with saline or valproic acid. The cub rats that were received from mothers who received saline on the postnatal day (PND) 12.5 were divided into control (CTL), exposure to tactile stimulation (TS) or whisker deprivation (WD), or TS plus WD (CTL–TS–WD) groups, and those whose mothers received valproic acid (Val) on the PND 12.5 were divided into valproic acid (Val), Valproic acid exposure to TS (Val–TS) or WD (Val–WD), or TS plus whiskers deprivation (Val–TS–WD) groups. TS was induced three times per day from PNDs 1 to 21 after birth (PNDs 1–21). All whiskers were trimmed from PND (1–21) in the WD groups. Data are shown as mean ± SEM. ^*^ *p* < .05, ^**^ *p* < .01, and ^***^ *p* < .001 (*n* = 7).

Multivariate ANOVA (sex × pretreatment × TS × WD) of PIST1 among female and male animals demonstrated a significant main effect for pretreatment [*F*(1, 96) = 7.22, *p =* .008] and TS [(*F*(1, 96) = 42.656, *p =* .0001)] but not for sex and WD variables. The (sex × pretreatment × TS × WD) interaction was not meaningful. Analysis of data for the main effect of the pretreatment variable demonstrated that in both sexes, the PIST1 decreased in the male‐Val group (about 23.27%) and the female‐Val groups (about 42.6%) compared with the corresponding control rats, which were significant for the female‐Val group compared to its CTL group (*p =* .002) but not for the male‐Val group compared to the CTL group (*p =* .07) (Figure 1).

In the male and female rats, sensory deprivation reduced the percentage of PIST1 by 17.35% and 21.62%, respectively, in the CTL–WD groups compared to the CTL groups, which was not significant. However, the performance was almost the same for the Val‐WD groups compared to the CTL–WD groups, showing a chance level (about 50%) for both sexes of the whisker‐deprived animals. Data analysis also showed that both sexes of the Val‐WD groups revealed an increase compared to the Val groups by 8.57% in males and 17.85% in females, which was insignificant in females.

Exposure to TS in the male‐CTL–TS group increased the percentage of PIST1 by 17.62% compared to the male‐CTL group, which was not significant. In female rats, this index was almost the same in the female‐CTL–TS group and the female‐CTL group. In the Val‐exposed rats, the percentage of PIST1 of the Val + TS groups compared to the Val groups (PIST1 percentage is 35.37% and 36.26%, respectively) showed a significant increase in both male (*p =* .001) and female (*p =* .016) rats.

No significant difference was observed between the CTL–TS–WD group and the CTL groups in male and female rats (8.08% and 5.43%, respectively), but the percentage of PIST1 in the Val–TS–WD group increased significantly compared to the Val group (*p =* .001 for males and *p =* .009 for females). Here, the performance of the Val–TS (1.81%) and the Val–TS–WD (1.65%) groups was almost the same. The results in this section showed that TS reduces the negative effects of sensory deprivation on the sociality behavior of rats under congenital valproic acid treatment.

#### 3.2.2 Preference index to Stranger 2 (PIST2)

**FIGURE 2:**
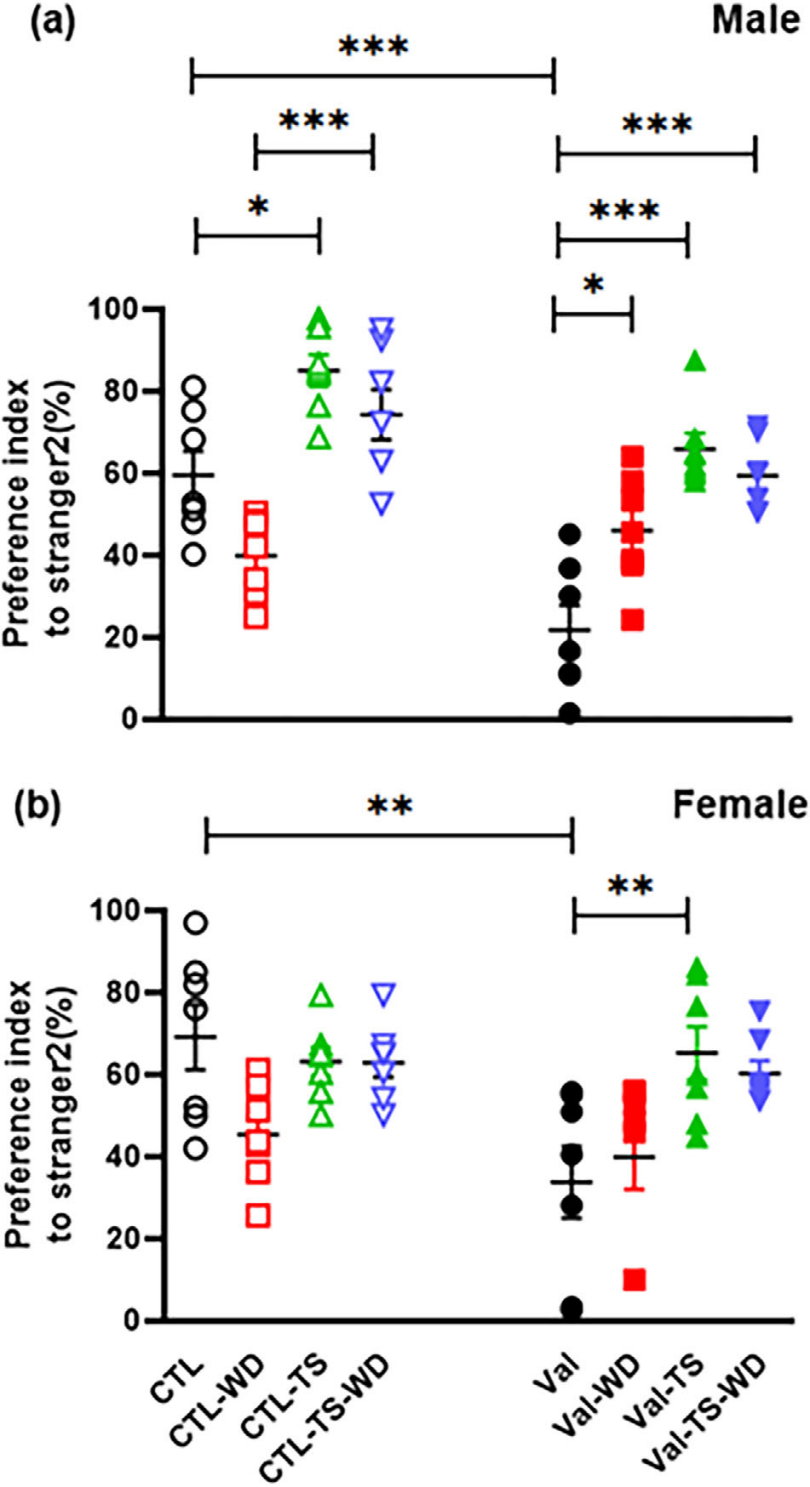
Novel phase of three‐chamber social test: The graphs show the preference index to Stranger 2 (Novel rat) versus Stranger 1 in both male (a) and female (b) offspring rats. The pregnant rats were prenatally treated with saline or valproic acid and were divided into the CTL and Val groups. The experimental rats were exposed to tactile stimulation (TS) and/or whiskers deprivation (WD). The other notifications are mentioned in Figure 1. Data are shown as mean ± SEM. ^*^ *p* < .05, ^**^ *p* < .01, and ^***^ *p* < .001 (*n* = 7).

Four‐way ANOVA (sex × pretreatment × TS × WD) of PIST2 among male and female rats showed a meaningful main effect for pretreatment [*F*(1, 96) = 26.34, *p* = .0001], TS [*F*(1, 96) = 70.66, *p* = .000] but not for WD [*F*(1, 96) = 3.12, *p* = .08] and sex. Interaction of sex × pretreatment × TS × WD was insignificant. Analysis of data for the main effect of the pretreatment variable revealed that in both sexes, the PIST2 was decreased by about 38% and 37% in the male‐Val group and the female‐Val groups compared to homologous control rats. As Figure 2 depicts, a significant decrease in the percentage of PIST2 was observed in the Val groups compared to the CTL groups in both males (*p =* .0001) and females (*p =* .001).

In both normal male and female rats, sensory deprivation reduces the percentage of PIST2 by 19.71% and 25.8%, respectively, in the CTL–WD groups compared to those of the CTL groups, but this reduction was not significant. However, the percentage of PIST2 in the male‐Val–WD group was not of any statistical significance compared to the male‐CTL–WD group (6.13% and 5.58%, respectively). It has been noted that a significant 24.2% increase in the PIST2 of the male‐Val–WD group compared to the male‐Val group (*p =* .02), but this index increased insignificantly by about 6.09% in female rats of the Val–WD group compared to the female‐Val group (Figure 2a,b).

TS induced a significant increase (25.46%) in the percentage of PIST2 in the male‐CTL–TS compared to that of the male‐CTL group (*p =* .015), but the statistical difference was not significant in female rats (8.02%). The percentage of PIST2 of the Val–TS groups of both sexes showed a significant increase compared to the homologous Val groups (for males [44.18%], *p =* .000, and for females [31.48%], *p =* .009).

The CTL–TS–WD rats in both sexes had no significant differences in PIST2 percentage compared to the control rats. In the Val–TS–WD group, a 37.65% increase in the PIST2 of male rats was significant in comparison with the Val group (*p =* .0001), but a 26% increase in the PIST2 of the female‐Val–TS–WD group compared to the female‐Val group was not significant (PIST2 percentage is 26.53%). Thus, the increase of PIST2 in autistic‐like rats under TS showed that TS reduces the negative effects of prenatal valproic acid exposure.

#### 3.2.3 Rearing and grooming

Four‐way ANOVA (sex × pretreatment × TS × WD) for the number of rats rearing in phase 1 of the three‐chamber social interaction test did not any meaningful main effect for sex, pretreatment TS, and WD variables. Moreover, the interaction of sex × pretreatment × TS × WD was not significant. In phase 2, there was a significant main effect for WD [*F*(1,96) = 6.47, *p* = .013) but not for sex, TS, and pretreatment. Interaction of sex × pretreatment × TS × WD was not significant. Four‐way ANOVA (sex × pretreatment × TS × WD) for the number of rats grooming in phase 1 of the three‐chamber social interaction test did not show any significant main effect for WD, sex, TS, and pretreatment. Interaction of sex × pretreatment × TS × WD was not significant. In phase 2, the main effect for the pretreatment was significant [*F*(1, 96) = 18.29, *p* = .0001) but not for sex, WD, and, TS. Interaction of sex × pretreatment × TS × WD was not significant (Table 3).

**TABLE 3:** Number of rats grooming and rearing in three‐chamber social interaction test

Groups									
	Test	CTL	CTL–WD	CTL–TS	CTL–TS–WD	Val	Val–WD	Val–TS	Val–TS–WD
Rearing in phase 1	Male	2.14 ± 0.51	2.7 ± 0.56	4.14 ± 0.98	4.00 ± 1.22	1.71 ± 0.52	5.14 ± 1.18^*^	4.43 ± 1.25	3.71 ± 1.23
	Female	2.14 ± 0.50	4.00 ± 0.95	2.57 ± 1.13	2.57 ± 0.99	1.71 ± 0.61	3.71 ± 0.94	4.71 ± 0.75	3.43 ± 0.95
Rearing in phase 2	Male	4.71 ± 1.19	4.71 ± 0.84	1.71 ± 0.68	4.71 ± 1.27	1.42 ± 0.53	4.00 ± 1.02	4.57 ± 0.97	3.00 ± 0.82
	Female	2.28 ± 1.06	3.57 ± 0.72	4.00 ± 0.96	2.71 ± 0.97	1.14 ± 0.26	3.71 ± 0.97	4.43 ± 0.78	2.86 ± 0.74
Grooming in phase 1	Male	2.14 ± 0.88	2.86 ± 0.70	3.14 ± 1.01	3.00 ± 0.22	1.29 ± 0.52	1.29 ± 0.29	1.71 ± 0.42	3.00 ± 0.61
	Female	2.14 ± 1.17	4.29 ± 1.17	3.14 ± 1.62	2.57 ± 0.99	2.71 ± 0.47	3.57 ± 0.81	4.57 ± 1.30	1.85 ± 0.77
Grooming in phase 2	Male	2.42 ± 0.57	3.57 ± 0.92	3.43 ± 0.48	3.29 ± 0.75	1.43 ± 0.53	2.57 ± 0.61	1.14 ± 0.45	4.00 ± 0.48
	Female	2.14 ± 0.51	4.29 ± 1.17	3.14 ± 1.63	2.57 ± 0.99	2.71 ± 0.47	3.57 ± 0.81	4.57 ± 1.31	1.86 ± 0.77

*Note*: Data are shown as mean ± SEM (*n* = 7).

Abbreviations: CTL, control; PND, postnatal day; TS, tactile stimulation; WD, whisker deprivation.

### 3.3 Novel texture recognition (NTR)

#### 3.3.1 Preference index to novel texture (PINT)

Four‐way ANOVA (sex × pretreatment × TS × WD) of preference index to novel texture (PINT) among male and female rats revealed a meaningful main effect for pretreatment [*F*(1, 96) = 12.53, *p =* .001), TS [*F*(1, 96) = 60.39, *p =* .000] but not for WD and sex. Interaction of sex × pretreatment × TS × WD was not meaningful. Interpreting for the main effect of the pretreatment variable revealed that the PINT in the male‐Val group (*p =* .0001) and the female‐Val group (*p =* .0001) showed a significant decrease compared to the homologous control rats by about 39% and 29.96%, respectively.

**FIGURE 3:**
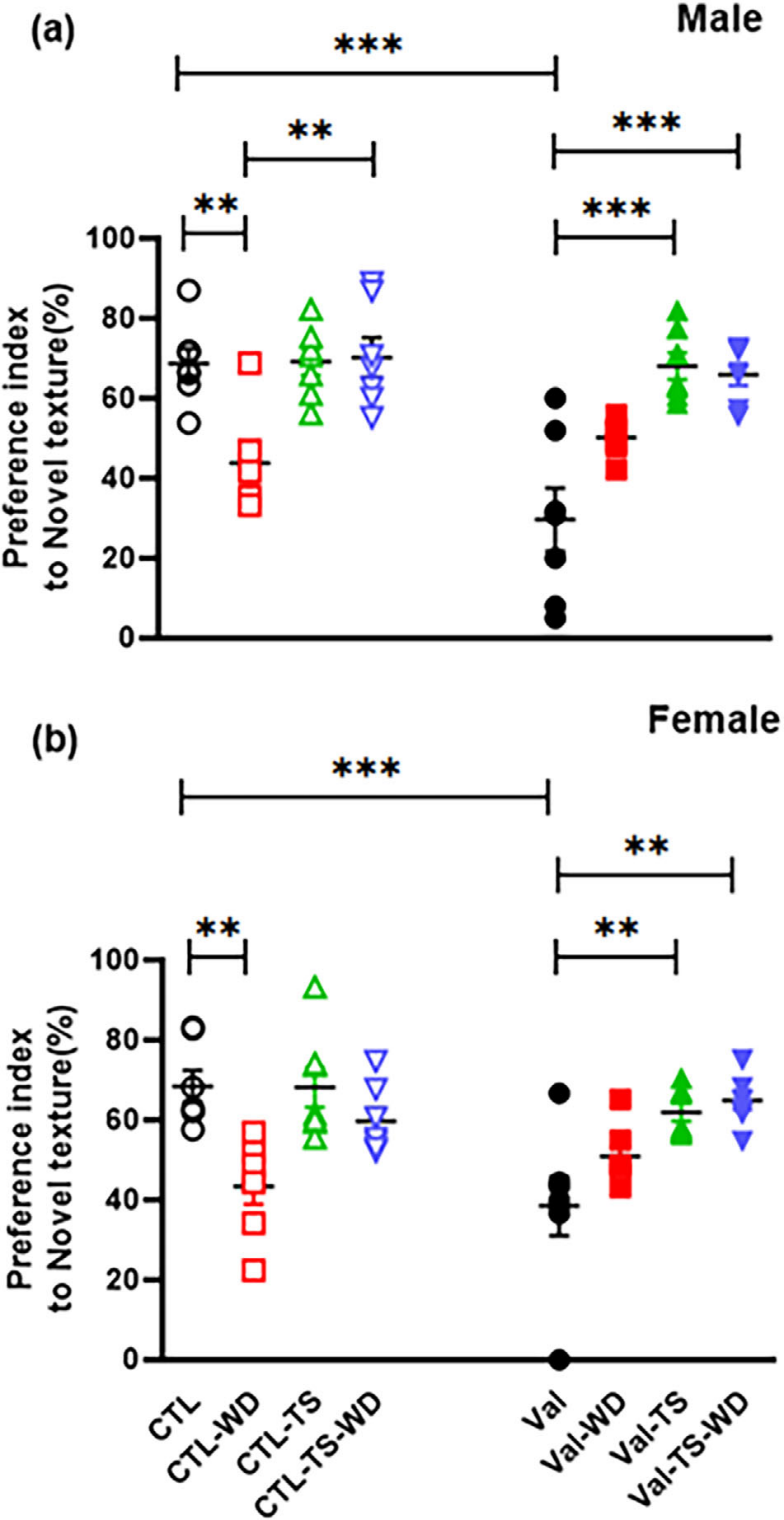
Novel texture recognition dependent on the somatosensory system: The graphs show the percentage of preference index to novel texture (PINT) versus old texture in both male (a) and female (b) offspring rats. The pregnant rats were prenatally treated with saline or valproic acid and were divided into the CTL and Val groups. The experimental rats were exposed to tactile stimulation (TS) and/or whiskers deprivation (WD). The other notifications are mentioned in Figure 1. Data are shown as mean ± SEM. ^*^ *p* < .05, ^**^ *p* < .01, and ^***^ *p* < .001 (*n* = 7).

As Figure 3 shows, sensory deprivation leads to a significant decrease in the percentage of PINT in the CTL–WD groups compared to their CTL groups in male (24.86%) and female (25.13%) rats (*p =* .006 and *p =* .006, respectively). In the autistic‐like rats, the percentage of PINT of the Val + WD groups was not significant in comparison with those of the Val groups in both male and female groups (males, 20.52% and females, 12.45%).

There was no statistically significant difference in the percentage of PINT between CTL–TS groups and CTL groups in both sexes, whereas the mean of PINT is about 70% for male and female rats under TS in normal conditions (female‐CTL–TS group). Interestingly, TS induced a significant increase in the Val + TS groups compared to the Val groups in both male (*p =* .0001) and female (*p =* .008) treated groups (for males, 38.4%, and females, 23.41%).

The compensatory effect of TS on the PINT% was also observed in the autistic‐like rats deprived of whiskers. As Figure 3 depicts, the percentage of PINT in the Val–TS–WD groups increased significantly compared to the Val groups in both males (*p =* .000) and females (*p =* .002). The percentage changes of PINT were 36.27% for males and 26.47% for females. Interestingly, in the normal rats, the compensatory effect of TS on the PINT% in the WD groups was observed in male rats. The 26.42% increase in the PINT% in the male–CTL–TS–WD group was significant compared to the male–CTL–WD group (*p* = .003), whereas in female rats, the PINT% did not show any significance (26.59%) in the CTL–TS–WD group, although a 16.3% increase was observed (Figure 3a,b). Therefore, in the normal and autistic‐like rats, TS improved the WD‐negative effects on the performance of rats for discrimination of textures.

#### 3.3.2 Rearing and grooming

The number of rearing and grooming of the rats that received TS and sensory deprivation did not reveal statistically significant compared to the control group in both sexes. Four‐way ANOVA (sex × pretreatment × TS × WD) for the number of rearing in both phases 1 and 2 of the NTR test did not show any meaningful main effect for WD, TS, sex, and pretreatment variables. Interaction of sex × pretreatment × TS × WD was also not significant.

In both phases 1 and 2 of the NTR, four‐way ANOVA (sex × pretreatment × TS × WD) for the number of grooming rats revealed no meaningful main effect for sex, TS, sex, and pretreatment. Interaction of sex × pretreatment × TS × WD was not significant (Table 4).

**TABLE 4:** Number of rats grooming and rearing in novel texture recognition test

Groups									
**Test**		**CTL**	**CTL–WD**	**CTL–TS**	**CTL–TS–WD**	**Val**	**Val–WD**	**Val–TS**	**Val–TS–WD**
**Rearing in phase 1**	**Male**	3.00 ± 0.53	2.000 ± 0.65	2.28 ± 0.47	3.29 ± 0.87	2.71 ± 0.71	3.14 ± 0.74	5.86 ± 1.53	5.43 ± 0.78
	**Female**	6.14 ± 1.99	5.57 ± 1.67	2.00 ± 0.43	2.00 ± 0.92	2.00 ± 0.75	3.14 ± 2.00	5.14 ± 1.80	3.71 ± 1.53
**Rearing in phase 2**	**Male**	4.85 ± 0.70	2.14 ± 0.45	2.42 ± 0.81	2.14 ± 1.01	2.85 ± 0.63	4.42 ± 1.54	6.00 ± 2.04	4.28 ± 0.89
	**Female**	5.85 ± 1.68	5.57 ± 1.25	4.57 ± 0.841	0.71 ± 0.35	3.85 ± 2.01	2.85 ± 1.47	6.71 ± 1.64	4.00 ± 1.54
**Grooming in phase 1**	**Male**	2.71 ± 0.42	1.00 ± 0.30	3.42 ± 0.92	2.14 ± 0.40	1.71 ± 0.60	2.71 ± 0.52	1.85 ± 0.50	3.14 ± 0.55
	**Female**	6.14 ± 1.99	5.57 ± 1.68	2.00 ± 0.93	2.00 ± 0.76	3.14 ± 2.01	5.14 ± 1.81	5.14 ± 1.81	3.71 ± 1.54
**Grooming in phase 2**	**Male**	4.86 ± 0.70	2.14 ± 0.46	2.43 ± 0.81	2.14 ± 1.01	2.86 ± 0.63	3. 43 ± 0.72	4.86 ± 1.68	4.29 ± 0.89
	**Female**	5.86 ± 1.68	2.00 ± 1.25	4.57 ± 0.84	0.71 ± 0.36	3.86 ± 2.02	2.86 ± 1.47	6.71 ± 1.64	4.00 ± 1.54

*Note*: Data are shown as mean ± SEM (*n* = 7).

Abbreviations: CTL, control; PND, postnatal day; TS, tactile stimulation; WD, whisker deprivation.

### 3.4 Nissl staining

#### 3.4.1 The percentage of degenerating neurons

**FIGURE 4:**
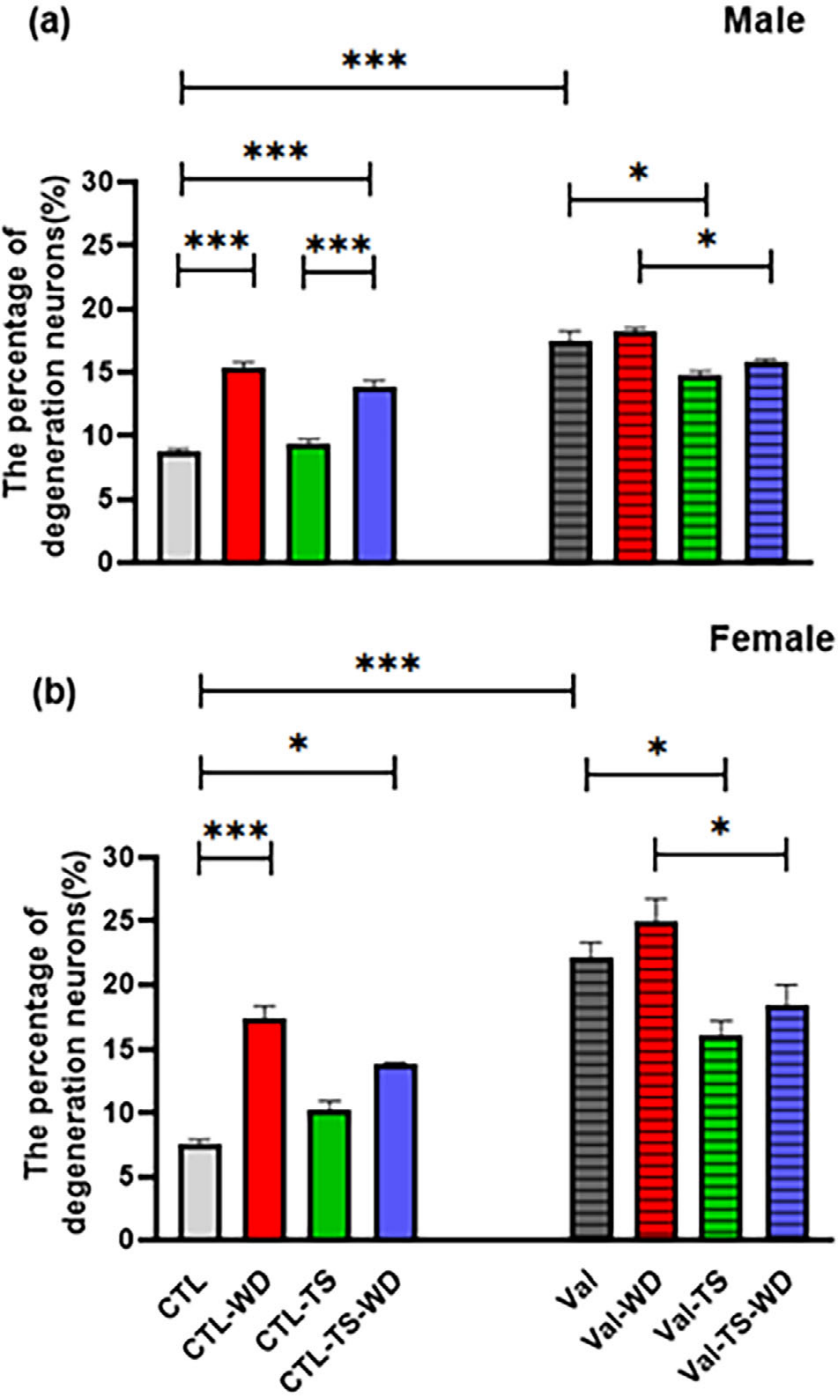
The percentage of degenerating neurons in both male (a) and female (b) offspring rats. The pregnant rats were prenatally treated with saline or valproic acid and were divided into the CTL and Val groups. The experimental rats were exposed to tactile stimulation (TS) and/or whiskers deprivation (WD). The other notifications are mentioned in Figure 1. Data are shown as mean ± SEM. ^*^ *p* < .05, ^**^ *p* < .01, and ^***^ *p* < . 001 (*n* = 3 rats/groups).

Four‐way ANOVA (sex × pretreatment × TS × WD) the percentage of degenerating neurons among male and female animals showed a significant main effect of sex [*F*(1, 32) = 26.45 *p =* .0001], pretreatment [*F*(1, 32) = 214.89, *p =* .0001], TS [*F*(1, 32) = 28.04, *p =* .0001], and WD [*F*(1, 32) = 80.70, *p* = .0001], whereas interaction of sex × pretreatment × TS × WD was insignificant. As Figure 4 depicted, a meaningful increase in degenerating neuron percentage in the Val groups compared to the CTL groups in both males (8.7%, *p =* .0001) and females (14.44%, *p =* .0001).

WD increased the percentage of degenerated neurons in the barrel field area, whereas there was a significant increase in the number of degenerated neurons in the CTL–WD groups in males (6.62%, *p =* .0001) and females (9.82%, *p =* .0001) compared to those of the CTL groups. In the autistic‐like rats, the difference between the percentages of degenerating neurons of the Val–WD groups compared to those of the Val groups in either of the sexes was not significant.

Although TS as a sensory stimulation did not significantly affect the number of degenerated neurons in the normal rats of either of the sexes in the CTL–TS groups compared to the CTL groups, in the autistic‐like rats of either of the sexes (for males, 0.57% and for females, 2.65%); the percentage of degenerating neurons in the Val + TS group showed a significant decrease compared the Val groups (*p =* .01 for males [2.69%] and *p =* .05 for females [5.98%]).

Interestingly, TS had no meaningful improving effect on the percentage of degenerating neurons in the whisker‐deprived normal rats. Here, the percentage of degenerating neurons of the male‐CTL–TS–WD groups (4.59%) showed a significant increase compared to male‐CTL–TS (*p =* .0001); the female‐CTL–TS–WD group did not show any significant difference in the percentage of degenerating neurons compared to the female‐CTL–TS group. But in autistic‐like rats, TS had a meaningful improving effect on the percentage of degenerating neurons in the whisker‐deprived rats. The results also showed that the Val–TS–WD groups significantly reduced the percentage of degenerating neurons compared to the Val–WD groups in both males (2.44%, *p =* .03) and females (6.44%, *p =* .02) (Figure 4). Briefly, TS improved the negative effects of WD on the percentage of degenerated neurons in normal and autistic‐like rats.

**FIGURE 5:**
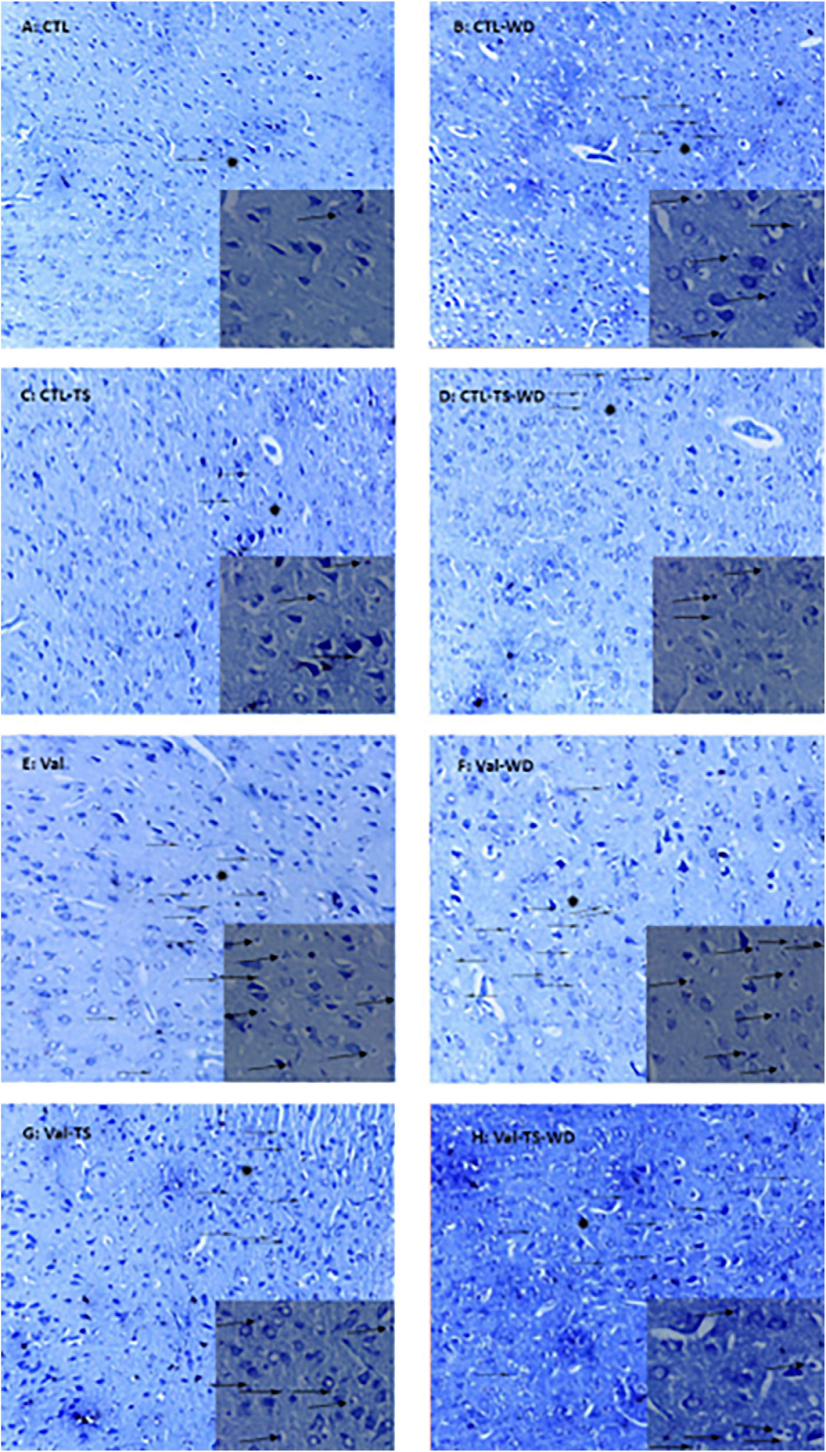
Micrographs sample of male barrel cortical cells stained by Nissl method. The pregnant rats were prenatally treated with saline (A‐D) or valproic acid (E‐H) and their offspring were divided into the control (CTL) and valproic acid (Val) groups. The experimental rats were exposed to tactile stimulation (TS) and/or whiskers deprivation (WD). The star sign is magnified in the small rectangles. The arrows show the degenerating neurons. Magnification ×100/400.

**FIGURE 6:**
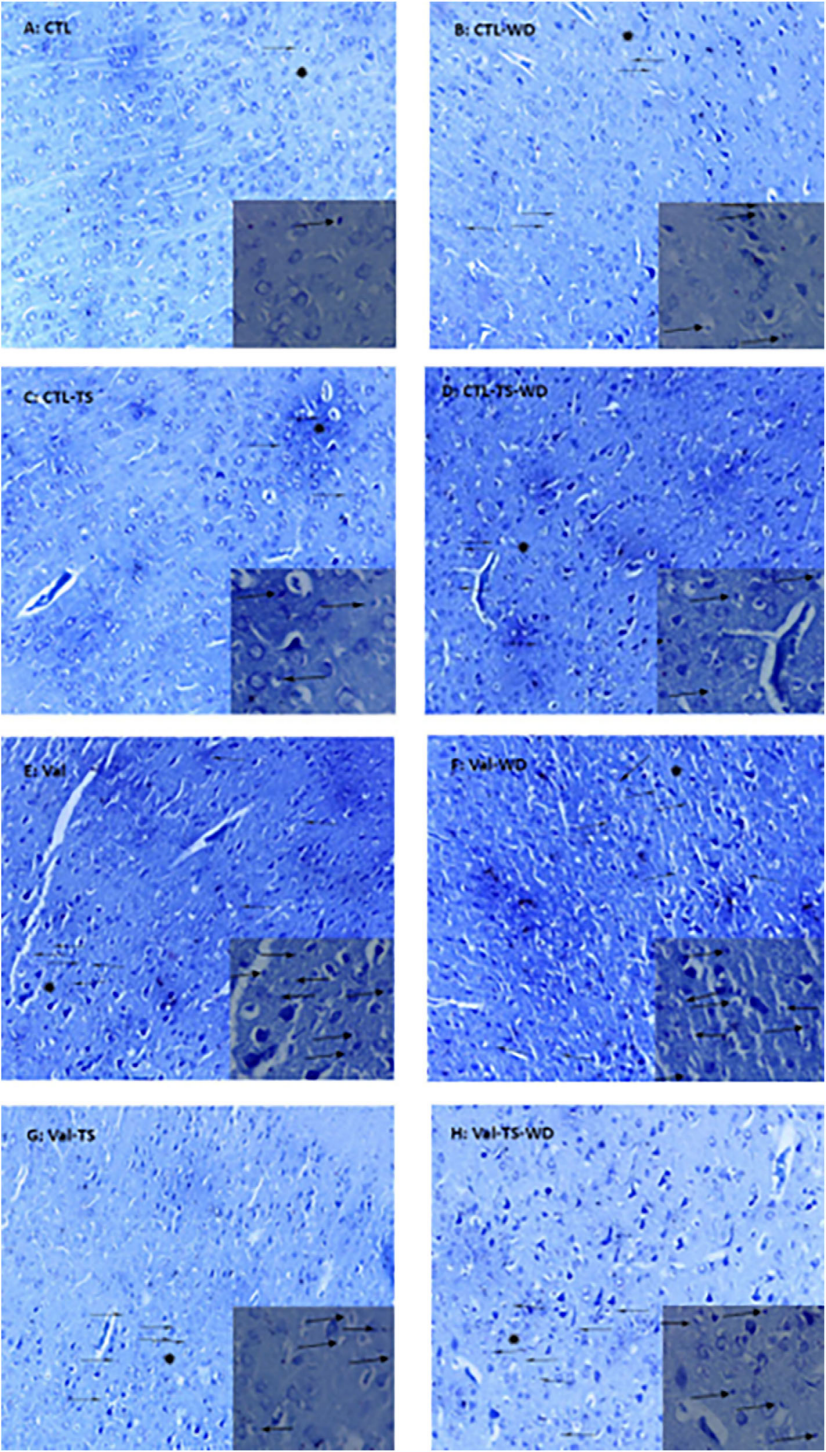
Micrographs sample of female barrel cortical cells stained by Nissl method. The pregnant rats were prenatally treated with saline (A‐D) or valproic acid (E‐H) and their offspring were divided into the control (CTL) and valproic acid (Val) groups. The experimental rats were exposed to tactile stimulation (TS) and/or whiskers deprivation (WD). The star sign is magnified in the small rectangles. The arrows show the degenerating neurons. Magnification ×100/400.

As Figures 5 and 6 show, in both males and females of the CTL–WD, CTL–TS–WD, Val, Val–WD, Val–TS, and Val–TS–WD groups, symptoms of neurodegeneration, including dense cytoplasm and dark cells, were revealed in the primary somatosensory cortex. The Val and Val–WD groups demonstrated a moderate‐to‐severe amount of degenerative neurons in males and females, respectively, which were improved by TS stimulation.

## 4 DISCUSSION

In the present study, the body weight gain of the Val‐treated rats (the Val, Val–WD, Val–TS, and Val–TS–WD groups) of both sexes was not significantly different compared with the control rats (the CTL, CTL–WD, CTL–TS, and CTL–TS–WD groups) in PND 1. It has been reported that the Val‐treated rats had no change in their body weight at PND 1 (Schneider & Przewłocki, 2005), whereas another study showed the body weight was significantly less in the Val‐exposed rats at PND 4 (Ruhela et al., 2019). At the PND 30, the body weight gain of the Val groups belonging to both sexes was not different statistically compared to the CTL rats, which was in accordance with the findings of others (Favre et al., 2013; Kim et al., 2013). However, the effect of period‐dependent valproic acid exposure has been investigated when valproic acid was injected into pregnant rats at embryonic days 7, 9.5, 12, and 15. No significant effect of period‐dependent valproic acid exposure on mothers’ body weight was reported in this study. It has been shown the body weight gain of rats exposed to valproic did not change until the 14th day of birth, but from the third week, the body weight gain decreased (Schneider & Przewłocki, 2005). The present results of body weight showed that this variable may be not a reliable factor for validating the rodent model of autism induced by valproic acid. So, other parameters such as tail bending (Saft et al., 2014) and the social behavior (Nygaard et al., 2019) of rats should be used. In our study, different degrees of tail abnormalities were observed in the rats treated prenatally with valproic acid.

The present study showed that both TS and WD did not alter the body weight gains of the juvenile male CTL–TS and CTL–WD groups compared with the CTL groups, whereas this was decreased significantly for females. The body weight gain of rats in the CTL–TS–WD groups reached the CTL groups on the PND 30. There are some studies that show TS (Haley et al., 2013; Richards, 2011) and WD (Afarinesh & Behzadi, 2018; Sun et al., 2010) did not affect the body weight of juvenile and adult rats. Age, gender difference, and the type of touch stimulus can be the reason for these contradictions.

Unlike the control groups (CTL–TS vs. CTL), TS caused an increase in the body weight gain of the Val–TS groups compared to the Val groups of both sexes (significant for females), which was in‐line with the findings of previous experiments (Akatsu et al., 2015; Caldji et al., 2000; Meerlo et al., 1999; Seiffe et al., 2022). The same result was obtained in the male‐Val–TS–WD rats under both TS and WD conditionings compared to the Val group, whereas the female‐Val–TS–WD group did not show any changes in the body weight gain compared to the Val group. It has been noted there was a significant increase in the weight of males in the Val–WD group compared to the Val group, but the weight of the females in the Val–WD group was less than that of the Val group. We did not know the reason for this difference in body weight gain in the Val‐treated rats (the Val–WD, Val–TS, and Val–TS–WD groups) and the control rats (the CTL–WD, CTL–TS, and CTL–TS–WD groups).

The present study showed in the three‐chamber social interaction test, prenatal treatment with valproic acid significantly reduced the percentage of preference index to Strangers 1 and 2 during sociability and novel social phases in both sexes of the Val groups compared with the CTL groups. It has been shown that prenatal injection of valproic acid reduces the sociability and novelty indices (Hara et al., 2017; Sabzalizadeh et al., 2022; Schiavi et al., 2019), which were in‐line with our study. It has been noted that the decrease in PIST2 in this test is an important criterion for induction of the ASD in rodents (Nygaard et al., 2019). Rats prenatally exposed to valproic acid also show deficits in the social domain due to deficits in social reward processing and the inability to properly understand and respond to social signals (Roullet et al., 2010). However, the exact mechanism for Val‐induced behavioral alterations is not known. This may be related to reduced neuronal activity by a blockage in sodium and calcium channels and by enhancement in the function of the inhibitory neurotransmitter γ‐aminobutyric acid (GABA) as a GABA transaminase inhibitor in the human brain (Kwan et al., 2001).

However, previous studies have shown that increased excitatory connectivity leads to microcircuitry hyperplasticity, overexpressed NMDA receptors, and hyperresponsiveness to stimulation, which makes a review of the neuropathology of autism necessary (Rinaldi et al., 2007, 2008). A novel view of the probable circuit pathology in autism is made possible by Intense World Syndrome. In this hypothesis, the excessive responsiveness of modular neocortical assemblies (e.g., minicolumns and columns) to stimulation causes them to become autonomous with runaway processing once triggered, a difficult process for higher brain regions to control, resulting in executive and other higher brain function deficits (Markram & Markram, 2010). The main cognitive pathologies believed to occur are hyper‐memory, hyper‐attention, and hyper‐perception (Silva et al., 2009). Many studies reported that prenatal exposure to Val causes many changes in neurotransmitters systems, including GABAergic (Martin & Manzoni, 2014), glutamatergic (Kim et al., 2013), serotonergic (Kinast et al., 2013), and dopaminergic (Schiavi et al., 2019).

The whiskers may also be used as an effective sensory organ in communication between rats. In the present study, we examined the possibility of this effect on their social interactions by prolonging rats’ deprivation of whiskers in prenatal Val rats. The performance of the Val + WD rats for PIST2 was based on the chance level in both sexes; nevertheless, in males of the Val + WD group, the PIST2 index increased compared to the Val group. The reason for the improvement in the function of autistic‐like rats under WD is not known. Perhaps WD reduces sensory input to the brain of the Val + WD groups and their exploration behaviors increased due to the thigmotaxis status of rats while they were not able to improve social interactions. Further studies are needed in this area. In this regard, it has been reported that abnormal social behaviors in whisker‐deprived rats are due to altered locomotor activity. These aberrant behaviors validate the hyperactivity and poor discrimination of early‐life sensory‐deprived animals (Lee et al., 2009). The increased social interactions found in the sensory‐deprived rats are also similar to the early‐life social‐deprived animals (Varlinskaya & Spear, 2008). However, in‐line with other studies (Afarinesh & Behzadi, 2017; Carvell & Simons, 1996; Lee et al., 2009), our behavioral and histological studies showed that WD decreased the ability of rats to discriminate texture due to deficits of the somatosensory cortex in both sexes.

The present study also showed that in the Val group, the percentage of preference index to novel texture decreased in both sexes compared to the CTL rats. In‐line with the present study, it has been demonstrated by a previous study that the ability to texture decreased in adult autistic‐like rats (Ho et al., 2009). A decreased sniffing time of novel objects in the object exploration tasks is also observed in male autistic‐like rats (Hara et al., 2012, 2017; Kang & Kim, 2015). It has also been reported that children with autism explore their environment less than control children (Bower, 1997). In humans, tactile defensiveness is a very common associated feature of ASD and Fragile X syndrome. An aversive and/or over‐reactive response to tactile stimuli considered harmless by most (e.g., the texture of clothing or light touch) is called tactile defensiveness. This phenomenon may also cause idiosyncratic eating habits due to the aversive texture or mouthfeel of many foods (Field et al., 2003; Harris, 2008).

The present study also showed that the number of degenerated cells of barrel cortical neurons of the Val groups increased in both sexes compared with the CTL groups, which was in‐line with other studies (Ahl, 1986; Kataoka et al., 2013). It has been noted that early prenatal Val exposure (E12.5) significantly decreases the number of Nissl‐positive cells in layers II–III and V of the prefrontal cortex and in layers IV–V of the somatosensory cortex, but not in layers II/III, whereas late prenatal Val exposure (E14.5) did not affect the number of Nissl‐positive cells in the somatosensory cortex (Kataoka et al., 2013).

Tactile sensory information from facial whiskers provides tactile information, including object texture and location, which appears to be computed in wS1 through the integration of motor and sensory signals. wS1 also directly controls whisker movements and contributes to learned, whisker‐dependent, goal‐directed behaviors (Petersen, 2019). Further integrative roles for complex neuronal circuits function in wS1 will probably be discovered through experiments involving more complex multi‐whisker‐dependent behaviors such as aperture discrimination and shape discrimination, as well as multisensory tasks such as navigation and social behaviors (Petersen, 2019). Interestingly, in primate evolutionary history, verbal communication is preceded by nonverbal communication (e.g., grooming tactile behavior interactions) (Petersen, 2019). It was noted that TS is effective as a treatment for improving emotional symptoms and communicative behavioral, social, and cognitive issues of ASD (Raza et al., 2015). TS also increases social dominance (Gromov, 2014), problem‐solving ability (Bernstein, 1952), and behavioral performance (Bernstein, 1952).

Our results showed that TS did not change the percentage of PIST1 in either sex in the CTL–TS rats, but there was a significant increase in the percentage of PIST2 in male rats of the CTL–TS group showing a gender difference. However, Richards (2011) reported that TS had almost no significant change in behavioral tests, including novel object test, T‐maze, and Y‐plus maze (Richards, 2011), which was in‐line with our results where TS did not influence the percentage of preference index to novel texture in the CTL–TS rats. Moreover, the percentage of degenerating neurons in the barrel field area did not show any significant change in either male‐ or female‐CTL–TS rats. Because behavioral tests such as texture discrimination tasks are simple to perform, the effect of TS may not be well highlighted in rats. Nevertheless, in the CTL–TS–WD group, the performance of rats under TS reached the level of the CTL group. Here, we showed that TS also improves the sociability interactions in the three‐chamber social interaction test and tactile discrimination of the Val–TS–WD rats compared with the Val group. In‐line with our behavioral study, the histology study also showed that the percentage of degenerating neurons decreased due to TS in the CTL–TS–WD and Val–TS–WD rats compared to the CTL group. However, deprivation after the onset of active sensing might elicit less harmful effects due to compensatory neuroplastic changes. Development of the whisker system in rats includes the early period of passive whisker touch (PNDs 1–8) before the onset of coordinated whisker movements that underlie active sensing. WD in pups during the first postnatal week is associated with weight loss. WD during PNDs 9–16 delayed self‐grooming (Smirnov & Sitnikova, 2019). It was noted that handling male mice for 12 days has no effect on adult body weight (Seiffe et al., 2022).

However, the present results revealed that prenatal treatment with valproic acid (the Val, Val–WD, Val–TS, and Val–TS–WD groups) did not change the number of groomings and rearings compared with the control (the CTL, CTL–WD, CTL–TS, and CTL–TS–WD groups) male and female rats in both three‐chamber social interaction and texture discrimination tests. It has been reported that the level of self‐grooming and rearing behaviors in Val‐treated mice was similar to that observed in control mice (Kang & Kim, 2015; Kim et al., 2008). However, the number of grooming movements is an indicator of anxiety‐like movements and coping with stress along with anxiety in rodents (O'Leary et al., 2013). Previous studies have shown that exposure to valproic acid during pregnancy leads to increased anxiety‐like behavior after birth (Moy et al., 2008; Yamaguchi et al., 2017). There is also a significant relationship between anxiety and restricted and repetitive behaviors in ASD patients (Mueller et al., 2013). The difference in the age, species, and type of maze and sensory stimulations may justify the lack of significant changes in these behaviors in our experiment compared to other studies. For example, it has been reported that exposure to an enriched environment in adolescent and adult Val and control group causes a significant increase in the number of rearings in the open‐box test (Schneider et al., 2006). Another study showed that handling male mice for 12 days from PNDs 22–34 improves sociability, repetitive behaviors (self‐grooming and Y‐maze alternation), and depression‐related behaviors (tail suspension and forced swim tests) and has no effect on adult body weight (Seiffe et al., 2022). Seiffe et al. (2022) also showed that the number of rearing had no changes between the handling group and the control group, but the duration of grooming increased in the Val group and significantly decreased in the handling group compared to the control group (Seiffe et al., 2022). Effects of prenatal stress and neonatal handling on rats were no significant difference in nursing posture, nest building, and self‐grooming (Akatsu et al., 2015).

## 5 CONCLUSION

Postnatal handling induces changes in adult anxiety and neuroendocrine reactivity (Meerlo et al., 1999). Prenatal exposure to valproic acid and WD is associated with peripheral afferent pathologies, especially the C‐tactile class of afferent thalamocortical gating systems (Chen et al., 2020; Esposito et al., 2013) that affect the distribution and regulation of tactile information in the cerebral cortex (Afarinesh & Behzadi, 2017; Catalano et al., 1995; Cho et al., 2017; Martin & Manzoni, 2014), structural patterns of the cortex such as lateral inhibition mediated by GABA and columnar organization (Jiao et al., 2006; Kim et al., 2013; Schierloh et al., 2004; Silva et al., 2009; Tatti et al., 2017), and so on, by prefrontal, somatosensory, or other cortical areas that play a role in cognitive functions. Treatment tactics, such as kangaroo care, massage, and TS, can enhance these effects. In particular, for neurodevelopmental disorders with a complex or unknown genetic origin, such as ADHD and ASD, sensory defensiveness, attention, and arousal can be modulated in older children by tactile‐focused therapeutic approaches, but further research is urgently needed on the mechanistic rationales and their efficacy (Cascio, 2010). Possible explanations include altered gene methylation (Mychasiuk et al., 2012), fibroblast growth factor‐2 (Comeau et al., 2007), brain‐derived neurotrophic factor (Sale et al., 2004), and increased production of neurotrophic factors, such as insulin‐like growth factor (Guzzetta et al., 2009), and altered endocrine function (Bock et al., 2005; Meaney et al., 1996). In addition, early experiments can alter synaptic organization by causing behavioral changes in infant and juvenile rats. An example is the effect of pre‐ and postnatal TS on play behavior (Muhammad et al., 2011; Muhammad & Kolb, 2011). Therefore, the extent and nature of play in juvenile rats modify the prefrontal cortex organization in adulthood (Bell et al., 2010). The present study showed that TS could compensate for the negative effects of congenital valproic acid exposure or WD in the sociability and novel social preference indices in the three‐chamber sociability interaction test, and the preference index to novel texture discrimination. Despite the improving role of TS on rat performance, no interaction between sensory deprivation and TS was observed as a synergistic or additive effect in autistic‐like rats. The tactile experience is important for the normal brain's functional, normal brain, and behavioral development (Olexová et al., 2016). Our results also showed that TS could help the normal whisker‐deprived rats to improve performance in tactile discrimination tests at behavioral and cellular levels. Despite the positive effects of TS on behavioral and histological parameters, previous studies have not been able to understand and prove the positive effects of tactile and sensory (Olexová et al., 2016), which may be due to differences in method or not eliminating the litter effect. Future studies can help to study mechanisms involving these effects.

## AUTHOR CONTRIBUTIONS

The authors confirm contribution to the paper as follows: study conception and design: Mohammad Reza Afarinesh. Data collection: Bi Bi Marzieh Ahmadi and Leila Jafaripour. Analysis and interpretation of results: Mohammad Reza Afarinesh and Bi Bi Marzieh Ahmadi. Draft manuscript preparation: Bi Bi Marzieh Ahmadi, Mohammad Reza Afarinesh, and Vahid Sheibani. All authors reviewed the results and approved the final version of the manuscript.

## CONFLICT OF INTEREST STATEMENT

The authors declare that they have no conflict of interest.

### PEER REVIEW

The peer review history for this article is available at https://publons.com/publon/10.1002/brb3.2993.

## Data Availability

The data that support the findings of this study are available from the corresponding author upon reasonable request.
